# Divergent roles of *hcp* genes in *Salmonella typhimurium* T6SS shape gut microbiota dysbiosis during infection

**DOI:** 10.1128/spectrum.02225-25

**Published:** 2026-02-27

**Authors:** Jia Liu, Jiao Liu, Chengjie Feng, Qinghua Zou

**Affiliations:** 1 Department of Microbiology and Infectious Disease Center, School of Basic Medical Sciences, Peking University Health Science Center 33133, Beijing, China; 2 Center of Medical and Health Analysis, Peking University Health Science Center 33133, Beijing, China; Tata Institute of Fundamental Research National Centre for Biological Sciences, Bangalore, Karnataka, India

**Keywords:** *Salmonella typhimurium*, type VI secretion system (T6SS), hemolysin-coregulated protein (Hcp), 16S rRNA sequencing, gut microbiota

## Abstract

*Salmonella enterica* subsp. *enterica* serovar *typhimurium* (*S. typhimurium*) is a facultative intracellular pathogen causing significant gastrointestinal infections in both humans and animals. The type VI secretion system (T6SS) plays a crucial role in its virulence, facilitating competition with host gut microbiota and promoting infection. While *S. typhimurium* possesses a single T6SS, it encodes three *hcp* genes, which are essential for its functionality. To investigate the non-redundant roles of each *hcp* gene, in this study, we used 16S rRNA sequencing to analyze gut microbiota in BALB/c mice after infection with wild-type (WT) *S. typhimurium* or three mutant strains (Δ*hcp1*, Δ*hcp2*, and Δ*hcp3*). Our findings revealed that *S. typhimurium* infection induced severe gut dysbiosis especially on the second day post-infection, which was characterized by a notable increase in Firmicutes and activation of the energy pathways. The deletion of each *hcp* gene led to distinct changes in bacterial taxa, underscoring the non-redundant function of each *hcp. Lactobacillus* showed significant effects in both strain main effects and strain-time interaction effects. Despite having only one T6SS, *S. typhimurium* achieves precise modulation of its functions through the divergent roles of its Hcp proteins, enabling efficient colonization and persistence in the host gut. These findings provide insights into the intricate mechanisms of bacterial adaptation and host-pathogen interactions, offering potential avenues for therapeutic interventions targeting T6SS-mediated dysbiosis.

**IMPORTANCE:**

*Salmonella enterica* subsp. *enterica* serovar *typhimurium* (*S. typhimurium*) is a facultative intracellular pathogen causing significant gastrointestinal infections in humans and animals. The type VI secretion system (T6SS) plays a crucial role in its virulence, facilitating competition with host gut microbiota and promoting infection. While *S. typhimurium* possesses a single T6SS, it encodes three *hcp* genes, which are crucial for its functionality and may exhibit non-redundant roles. In this study, we used 16S rRNA sequencing to analyze gut microbiota in BALB/c mice after infection with wild-type (WT) *S. typhimurium* or mutant strains (Δ*hcp1*, Δ*hcp2*, Δ*hcp3*). Our findings revealed that the deletion of individual *hcp* genes led to distinct bacterial taxa changes, underscoring the non-redundant functions of each *hcp*. Despite having only one T6SS, *S. typhimurium* achieves precise modulation of its functions through the divergent roles of its Hcp proteins, enabling efficient colonization and persistence in the host gut.

## INTRODUCTION

*Salmonella enterica* subsp. *enterica* serovar *typhimurium* (*S. typhimurium*) is a facultative intracellular pathogen that infects a wide range of host species, causing diseases such as gastroenteritis in humans and systemic infections in animals (1). As a well-established model organism for studying *Salmonella* pathogenesis, *S. typhimurium* has been extensively investigated to elucidate its virulence mechanisms and interactions with host cells (2). Central to its pathogenicity is the type VI secretion system (T6SS), a sophisticated protein delivery apparatus that enables the bacterium to translocate effector proteins into host cells or competing microbes (3). The T6SS enhances bacterial survival and fitness by facilitating intracellular replication, promoting immune evasion, and mediating inter-bacterial competition.

A critical component of the T6SS machinery is the hemolysin-coregulated protein (Hcp), which plays a pivotal role in the secretion process (4). *S. typhimurium* encodes three distinct *hcp* genes—*hcp1*, *hcp2*, and *hcp3*—with *hcp1* and *hcp2* located in the T6SS cluster and *hcp3* outside the cluster. These genes are differentially expressed under various environmental conditions and contribute to the bacterium’s adaptability and virulence (5). Investigating the roles of *hcp* genes is essential to fully understand the functional versatility of T6SS in *Salmonella* pathogenesis.

Thibault et al. demonstrated that *S. typhimurium* utilizes T6SS to kill members of the normal microbiota, such as *Klebsiella oxytoca*, thus facilitating bacterial colonization and infection in mice (3). Similarly, Pezoa et al. investigated the specific roles of T6SS encoded by SPI-6 and SPI-19 in chicken and mouse infections, respectively, highlighting their importance in host-pathogen interactions (6). However, these studies primarily focused on a limited number of bacteria in the gut, which does not adequately capture the broader ecological shifts and functional dynamics of the gut microbiota influenced by T6SS activity.

The 16S ribosomal RNA (rRNA) sequencing technique has emerged as a cornerstone method to study microbial communities due to its precision and efficiency (7). This technique targets the 16S rRNA gene, which contains both conserved and variable regions. The conserved regions facilitate universal primer binding, while the variable regions provide taxonomic information, enabling the identification and classification of diverse microbial taxa. Unlike traditional culture-based methods, 16S rRNA sequencing allows for the comprehensive analysis of both culturable and unculturable microorganisms, thus overcoming significant limitations in microbial ecology research (8). Ke Ding and colleagues explored the role of ClpV, a key ATPase in the T6SS machinery (9). While their study incorporated some analyses of gut microbiota, the depth of data mining for 16S rRNA sequencing results was insufficient, leaving gaps in understanding the broader impacts of T6SS on gut microbial ecology and host-pathogen interactions.

To address these gaps, our study utilized 16S rRNA sequencing to investigate the gut microbiota of BALB/c mice infected with four different *S. typhimurium* strains: wild-type, Δ*hcp1*, Δ*hcp2*, and Δ*hcp3*. Comprehensive analyses of the sequencing data were conducted to explore the community composition, alpha and beta diversity, and differential taxa across the groups. Furthermore, functional prediction of the gut microbiota was performed to provide insights into the potential biological implications of *hcp* gene deletions on microbial communities.

## MATERIALS AND METHODS

### Mouse infection assays

BALB/c female mice (6–8 weeks old, weighing 17.4 ± 0.9 g, SPF grade) were purchased from the Experimental Animal Center of Peking University Health Science Center. No prior procedures were performed on the animals before the start of the study. Environmental conditions were maintained at a temperature of 20–24°C with 40%–60% relative humidity, and a 12-hour light/dark cycle. Animals were provided with autoclaved corn cob bedding, which was changed twice weekly. Sterilized water and standard laboratory chow were available *ad libitum* throughout the study. No additional environmental enrichment was provided during the infection period in order to minimize potential microbial or behavioral confounding factors.

The 32 mice were randomly divided into 4 groups (8 mice in each group) and acclimated for 4 days prior to the infection. During the acclimation period, the mice were gently massaged daily in the afternoon to stimulate defecation. Fecal samples were promptly collected into sterile Eppendorf tubes, with one sample collected each day (N1, N2, N3, N4) before infection. Following a 4-h fasting period, mice were gavaged with 5% sodium bicarbonate solution to neutralize gastric acid. After 30 min, each mouse was orally administered 200 µL of a bacterial suspension (10⁶ CFU/mL). The bacterial strains used in this study are wild-type 14028s and three *hcp* mutants (Δ*hcp1*, Δ*hcp2*, and Δ*hcp3*). All strains were cultured in antibiotic-free LB broth under standard conditions. Oral gavage was performed using sterile flexible feeding needles by trained personnel to reduce injury or stress. No unexpected adverse events were observed during the course of the study.

Subsequently, four mice from each group were sacrificed after infection by cervical dislocation on day 2 and day 5, respectively. Liver, spleen, intestine, and intestinal contents were rapidly collected. Contents from the colon and cecum were stored at −80°C for subsequent 16S rRNA sequencing. Liver, spleen, and colon contents were homogenized, diluted, and plated onto MacConkey and PSE agar plates to classify and quantify bacterial populations.

### DNA extraction and PCR amplification of intestinal contents

Intestinal content samples were placed into sterile 2 mL centrifuge tubes, followed by the addition of 1 × PBS solution. The mixture was vortexed thoroughly and centrifuged at 10,000 rpm for 3 min at room temperature. The supernatant was discarded, and the tubes were inverted on absorbent paper for 1 min to ensure the complete removal of residual liquid. DNA was extracted from the samples using the E.Z.N.A Mag-Bind Soil DNA Kit (OMEGA), and the integrity and concentration of the DNA were verified by gel electrophoresis.

The genomic DNA was accurately quantified using the Qubit 3.0 DNA Assay Kit to determine the appropriate amount of DNA for PCRs. The first round of PCR amplification was performed in a 30 µL reaction mixture, which included 15 µL of 2× Hieff Robust PCR Master Mix, 10–20 ng of sample DNA, and 1 µL each of 10 µM forward and reverse primers (Bar-PCR primer F and Nobar_805R). The PCR program was as follows: 94°C for 3 min; 5 cycles of 94°C for 30 s, 45°C for 20 s, 65°C for 30 s; 20 cycles of 94°C for 20 s, 55°C for 20 s, 72°C for 30 s; 72°C for 5 min; hold at 10°C. For the second round of amplification, 20–30 ng of the first-round PCR product was added to a 30 µL reaction mixture containing 1 µL each of 10 µM forward and reverse primers (Index-PCR primer R and Nobar_341F). The PCR program was as follows: 95°C for 3 min; 5 cycles of 94°C for 20 s, 55°C for 20 s, 72°C for 30 s; 72°C for 5 min; hold at 10°C. The library size was assessed using 2% agarose gel electrophoresis. To ensure uniform clustering and high-quality sequencing data, the library concentration was quantified using a Qubit 3.0 fluorometer. The libraries were subjected to high-throughput metagenomic sequencing using the Illumina MiSeq platform (performed by Sangon Biotech Co., Ltd., Shanghai, China).

### Sequencing data analysis and statistical analysis

The PE reads generated by Illumina MiSeq sequencing were assembled based on overlapping regions and assigned to samples. Sequence quality was assessed, and low-quality reads were filtered out. Operational taxonomic units (OTUs) were clustered at 97% sequence similarity, followed by taxonomic classification and analysis. Taxonomic classification was performed using the Ribosomal Database Project (RDP) classifier against the RDP database. The analysis included rarefaction curves, Ace and Chao richness indices, and Shannon and Simpson diversity indices to evaluate alpha diversity. Based on taxonomic information, community structure was analyzed at different taxonomic levels. Further analyses included beta diversity assessment, comparative group analysis, significance testing for differential taxa, and functional prediction, incorporating phylogenetic and community composition data.

Alpha diversity indices of gut microbiota were expressed as mean ± standard deviation (*x* ± *s*). Statistical analyses were performed using R studio. One-way analysis of variance (ANOVA) was used for comparisons among multiple groups, followed by *t*-test for pairwise comparisons. A *P*-value < 0.05 was considered statistically significant. PICRUSt was used to infer the metagenomic functional content from 16S rRNA gene sequences based on the Greengenes database and to generate Kyoto Encyclopedia of Genes and Genomes (KEGG) Ortholog (KO) abundance tables. The abundance tables were normalized by *z*-score transformation and visualized using the pheatmap package in R (version 4.3.1).

## RESULTS AND DISCUSSION

### Confirmation of *S. typhimurium* strains and characterization of infected mice

All *S. typhimurium* strains used in this study—WT, three *hcp* deletion mutants, and their corresponding complemented strains—were confirmed by PCR. As shown in Fig. S1, agarose gel electrophoresis of PCR products targeting the homologous regions confirmed the intact presence of *hcp1* and *hcp3* in the wild-type background. PCR amplification of the deletion loci produced the expected band patterns (Fig. S2), demonstrating the precise removal of the respective *hcp* genes in each mutant strain. Fig. S3 confirmed the successful construction of the complemented strains. The functional activity of Hcp1 has been demonstrated previously in our published study (5), which showed that the Δ*hcp1* mutant exhibits significantly reduced motility, and that motility is restored to wild-type levels upon complementation. To demonstrate that Hcp2 and Hcp3 are active, we performed amoeba co-culture assays. Δ*hcp2* and Δ*hcp3* mutants exhibited markedly reduced attachment to and internalization by amoebae, while the number of extracellular bacteria increased compared with WT (Fig. S4 and S5 in the supplemental material). Interestingly, the double mutant (Δ*hcp2*Δ*hcp3*) reverted to WT-like levels, suggesting non-redundant but complementary activities of the two Hcps. In our previous study (5), we demonstrated that *hcp* mutant strains lose inter-bacterial competitive ability when co-cultured with specific bacterial targets. In this study, we further investigate the effects of these mutants on a more complex gut microbiota in mice.

**Fig 1:**
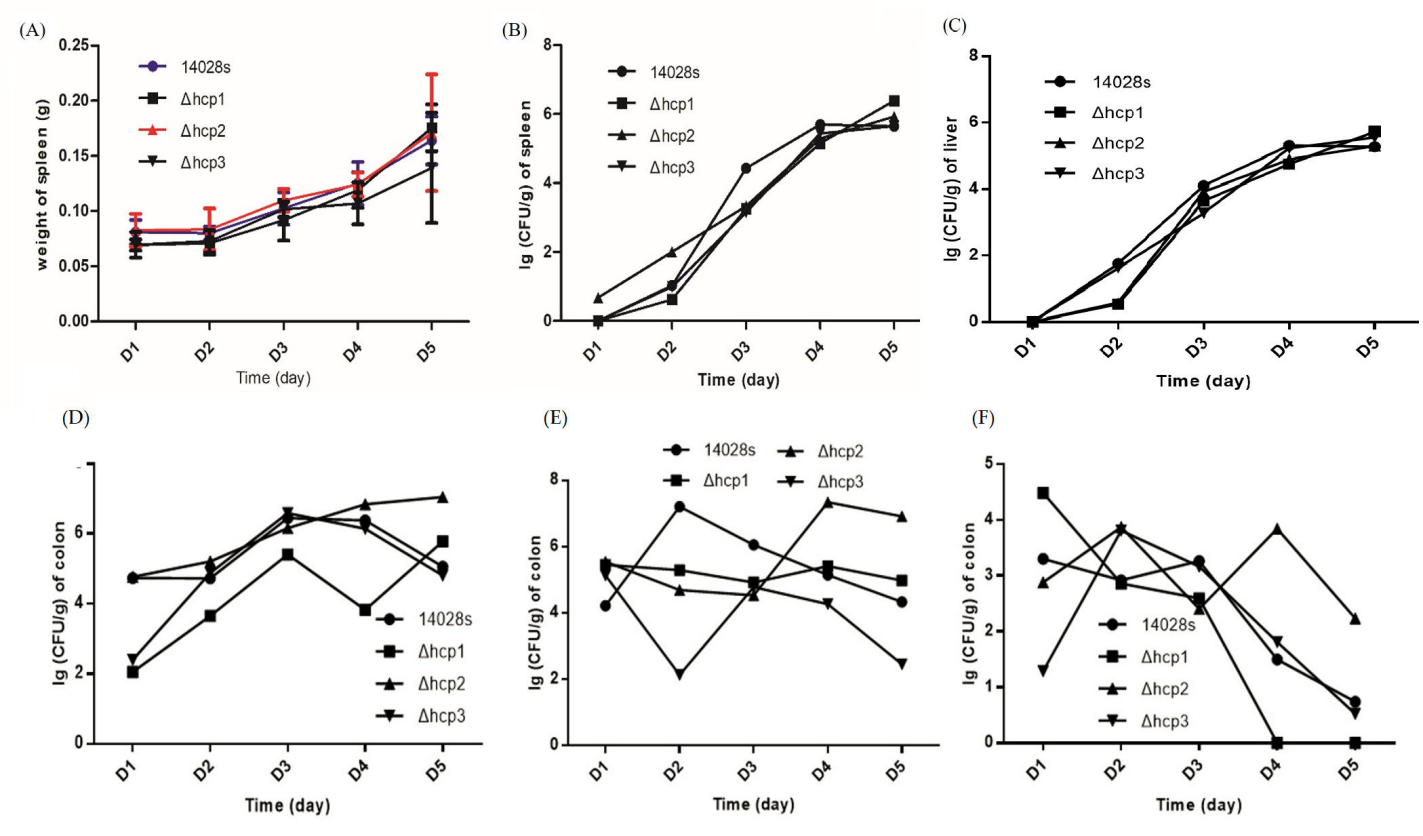
Bacterial loads in various organs of mice infected with wild-type *S. typhimurium* and its three hcp mutant strains over the course of infection. (**A**) Spleen weight and (**B**) *Salmonella* load in spleen. (**C**) *Salmonella* load in liver. The bacterial loads of *Salmonella* (**D**), *Enterococcus* (**E**), and *Escherichia coli* (**F**) in the colonic contents. Infection led to an increase in spleen weight and bacterial loads in spleen and liver across all groups.

Female BALB/c mice were selected because they are a well-established small animal model for *S. typhimurium* gastrointestinal infection and host–microbiota interaction studies. To evaluate the physiological effects of wild-type *S. typhimurium* and its three *hcp* mutant strains (Δ*hcp1*, Δ*hcp2*, Δ*hcp3*) on infected mice, body weight, spleen weight, bacterial load, and gut microbiota composition were monitored at different time points post-infection. The changes in body weight of the mice after infection are shown in Fig. S6 in the supplemental material. Fig. 1 presents the spleen weight and *Salmonella* load in the spleen. Infection led to an increase in spleen weight across all groups (Fig. 1A). The bacterial loads in spleen (Fig. 1B) and liver (Fig. 1C) followed a similar trend, with no significant differences among the groups. Fig. 1DEF details the bacterial populations within the colonic contents, including *Salmonella*, *Enterococcus*, and *Escherichia coli*, indicating the distinct patterns of these bacterial populations in the gut microbiota among the different groups. Inspired by these results, we performed 16S rRNA sequencing to comprehensively analyze microbial diversity, community composition, and functional predictions.

### Microbial diversity

To ensure the reliability of the sequencing data, rarefaction curve analysis was performed to evaluate the sequencing depth, see Fig. S7. The rarefaction curves gradually plateaued with increasing sequencing depth, indicating that the data volume was adequate to represent the microbial diversity and composition of the samples comprehensively. The validity of sample grouping was assessed by Anosim (analysis of similarities) and Adonis (permutational multivariate analysis of variance) analyses (data not shown). Both algorithms confirmed the appropriateness of the experimental grouping and suggested that the observed microbial community differences were driven by the infection with wild-type or mutant strains of *S. typhimurium*.

**Fig 2:**
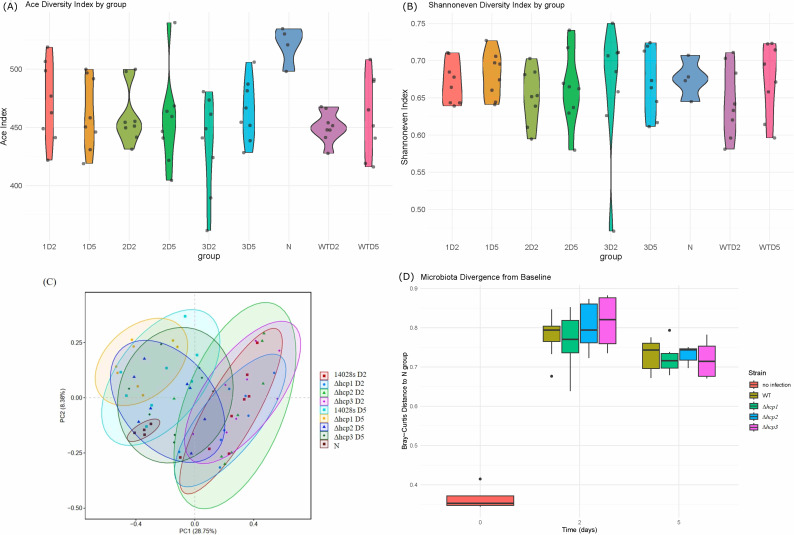
Alpha and beta diversity analysis of the gut microbiota in infected mice. Violin plots of (**A**) ACE index and (**B**) Shannoneven index values across groups. (**C**) PCA plot of microbiota composition for all groups. (**D**) Bray–Curtis distances between post-infection samples and the baseline group. While the infection impaired the overall richness of the gut microbiota, the balance of species abundance remained relatively stable. *S. typhimurium* infection induced severe gut dysbiosis especially on the second day post-infection.

To evaluate the impact of *S. typhimurium* infection and its mutant strains on gut microbiota diversity, two key metrics of alpha diversity were analyzed: the ACE index, which assesses species richness, and the Shannoneven index, which reflects the evenness of species distribution. The results of Wilcoxon rank-sum tests for group comparisons are summarized in Tables S1 and S2. As shown in Fig. 2A, violin plots of ACE index values indicate that the pre-infection group (N group) exhibited significantly higher species richness compared to the post-infection groups, irrespective of the bacterial strain used. This suggests that *S. typhimurium* infection substantially reduced the richness of gut microbiota. However, no significant differences were observed in ACE values among the infected groups, indicating that the richness was similarly affected across wild-type and mutant strains. Fig. 2B displays violin plots of Shannon even index values across groups. Unlike species richness, the evenness of species distribution was not significantly altered by infection with either the wild-type or mutant strains. This observation suggests that while the infection impaired the overall richness of the gut microbiota, the balance of species abundance remained relatively stable.

Beta diversity analysis was also performed to evaluate the compositional differences in gut microbiota across groups over time. Fig. 2C presents the principal component analysis (PCA) of microbiota composition for all groups. The results indicate a distinct shift in gut microbial communities on the second day post-infection with the wild-type *S. typhimurium* strain and its mutant strains, compared to the pre-infection state. By the fifth day post-infection, the gut microbiota appeared to gradually return to a composition closer to the pre-infection state. This observation aligns with expectations, as a low-dose infection model was employed in this study. To quantify the resilience of the gut microbiota following infection, we analyzed the degree to which the microbial community structure of each group recovered from day 2 to day 5, relative to the pre-infection baseline (N group). As shown in Fig. 2D, the Bray–Curtis distances between post-infection samples and the baseline group were compared at two time points. Notably, the wild-type and three *hcp* mutant groups showed a significant reduction on day 5, indicating a measurable restoration of the microbial community structure. In addition, the Samplecorrplot (Fig. S8) indicated the similarity and relativity among samples, which also revealed the subsequent recovery by the fifth day post-infection.

### Structure of bacterial communities

**Fig 3:**
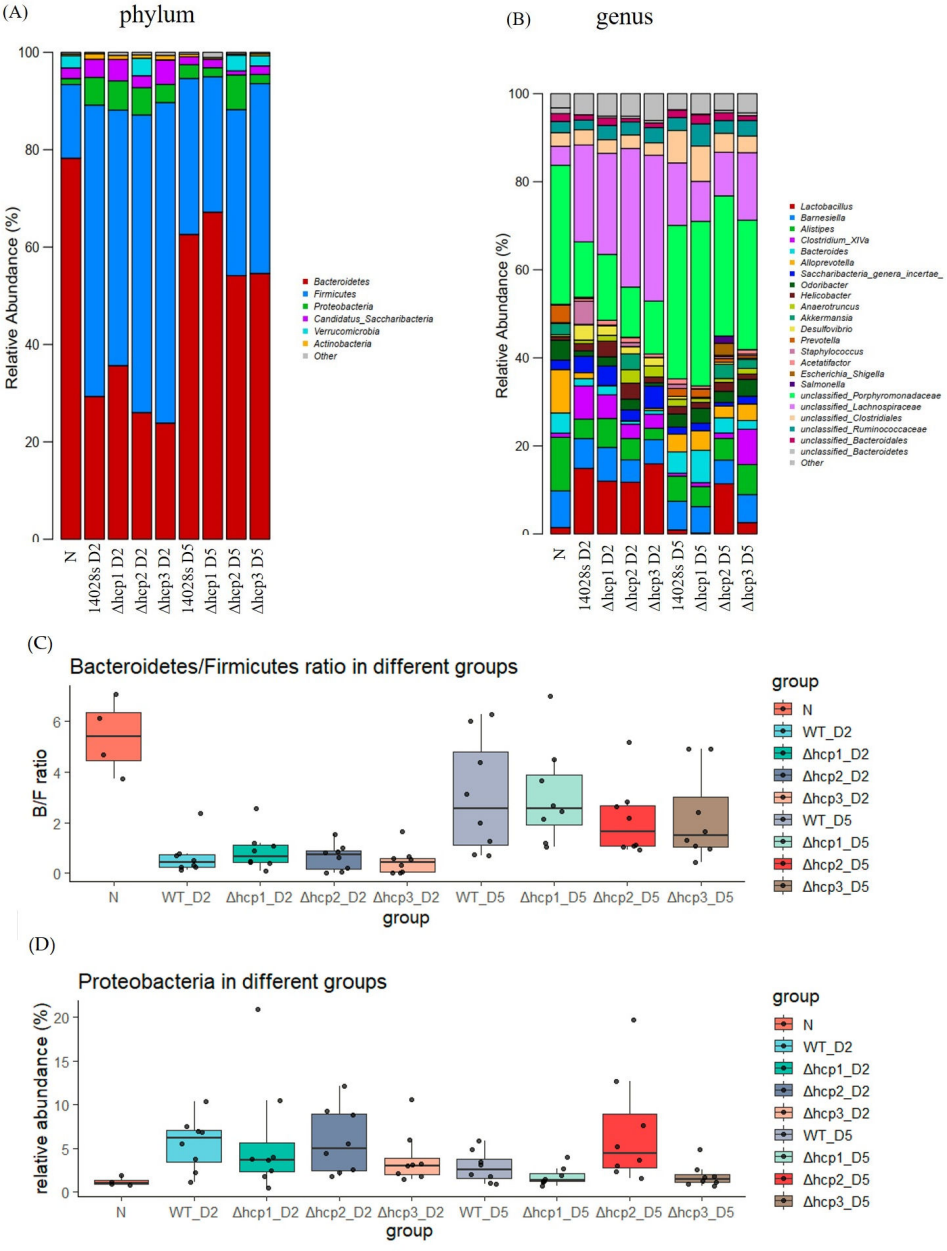
Structure of bacterial communities of the gut microbiota at phylum (**A**) and genus (**B**) levels in infected mice. (**C**) Bacteroidetes/Firmicutes ratio in different groups. (**D**) Proteobacteria in different groups. Bacteroidetes/Firmicutes (B/F) ratio was significantly decreased, and proteobacteria was increased on the second day post-infection.

To investigate the compositional changes in the gut microbiota across different groups, stacked bar plots of dominant taxa were generated at the phylum and genus levels (Fig. 3A and B). Notably, the Bacteroidetes/Firmicutes (B/F) ratio, a widely recognized indicator of gut microbiota dysbiosis, was significantly decreased on the second day post-infection compared to the pre-infection state at phylum level, as shown in Fig. 3C. By the fifth day post-infection, the B/F ratio returned toward pre-infection levels, reflecting a recovery of microbial community structure, which is in line with the Bray–Curtis distances (Fig. 2D). Statistical analysis confirmed the significance of these changes (data not shown). Firmicutes are known to include species that produce short-chain fatty acids (SCFAs), such as butyrate, which support intestinal barrier integrity and regulate immune responses (10). Conversely, Bacteroidetes are associated with polysaccharide metabolism and have been implicated in maintaining immune homeostasis (11). A decrease in Bacteroidetes relative to Firmicutes, as observed in this study, may disrupt the balance of SCFA production and host-microbe interactions, thereby exacerbating gut inflammation and promoting pathogen persistence. A similar phenomenon was also observed at the levels of class, order, and family. Besides the B/F ratio, proteobacteria was also increased on the second day post-infection, indicating the impaired function of intestinal barrier (12) (Fig. 3D).

At the genus level (Fig. 3B), wild-type *S. typhimurium* infection on day 2 exhibited a significant increase in *Escherichia-Shigella*, suggesting inflammation-associated dysbiosis. In contrast, the Δ*hcp1*, Δ*hcp2,* and Δ*hcp3* groups showed lower *Escherichia-Shigella* levels, implying a milder disruption of gut homeostasis.

### Differential taxa identified by LEfSe analysis, Wilcox, and ANOVA test

**Fig 4:**
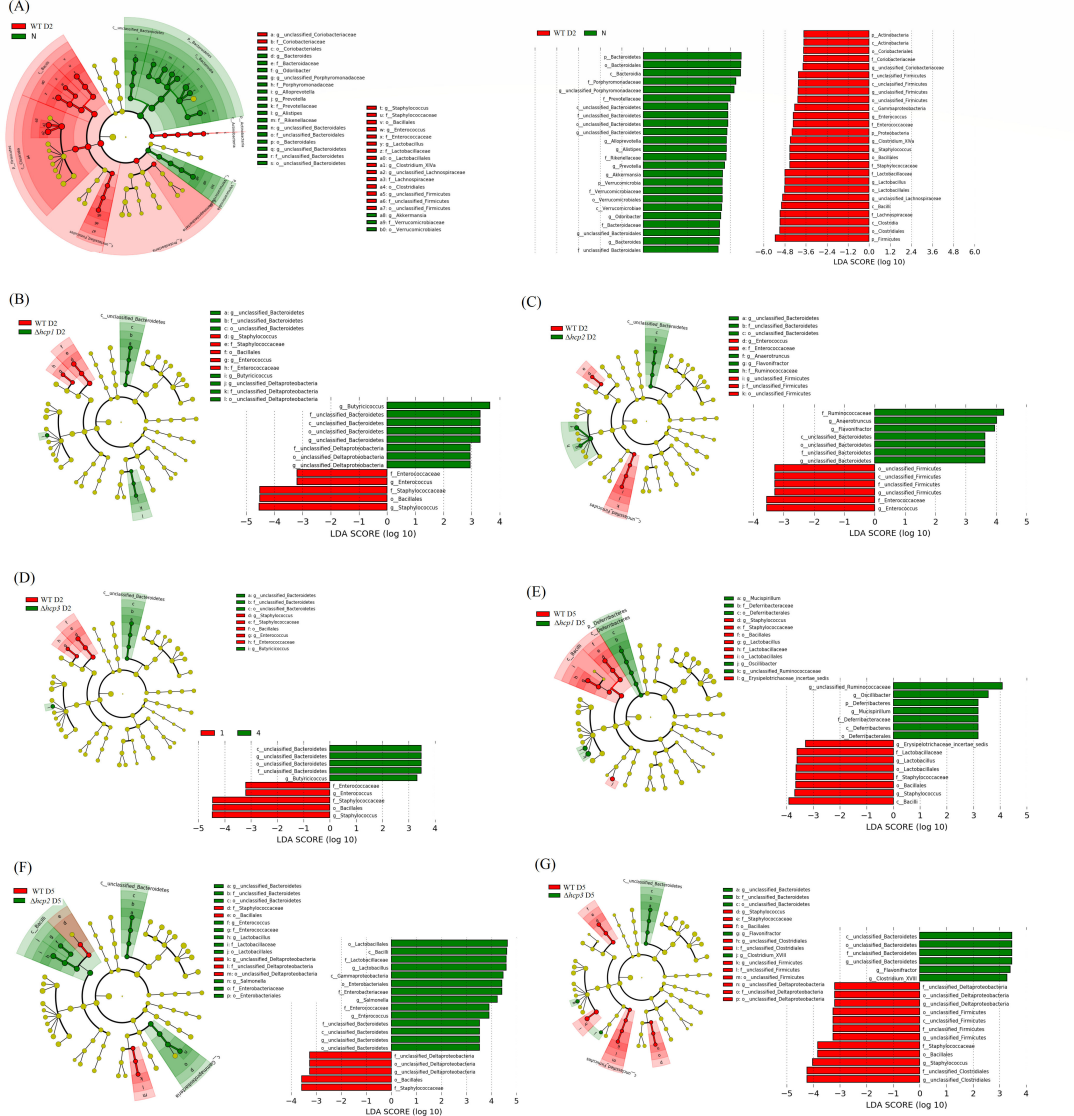
Differential taxa identified by LEfSe analysis. (**A**) The cladogram of mice before infection (N group) and two days post-infection with the wild-type *S. typhimurium*. LEfSe cladograms on day 2 after infection with Δhcp1 (**B**), Δhcp2 (**C**), and Δhcp3 (**D**) strains compared to wild-type *S. typhimurium*. LEfSe cladograms on day 5 after infection with Δhcp1 (**E**), Δhcp2 (**F**), and Δhcp3 (**G**) strains compared to wild-type *S. typhimurium*. While deleting any single hcp gene does not completely abolish the functionality of the T6SS, each hcp gene appears to have specific contributions to the microbial shifts mediated by *S. typhimurium*.

Figure 4A shows a cladogram highlighting the taxonomic differences between the gut microbiota of mice before infection (N group) and 2 days post-infection with the wild-type *S. typhimurium*. Taxa within the phylum Bacteroidetes, including several genera known for their roles in maintaining gut homeostasis, were significantly reduced after infection. Similarly, members of the phylum Verrucomicrobia, such as *Akkermansia*, which are associated with gut barrier integrity and anti-inflammatory effects, showed a notable decrease (13). Conversely, the relative abundance of Firmicutes, particularly taxa within the classes Clostridia and Bacilli, was significantly elevated. This increase may reflect the proliferation of opportunistic bacteria in response to altered gut conditions during infection, including nutrient availability and immune modulation. Additionally, the phylum Proteobacteria, which includes many potential pathogens or pathobionts, also expanded significantly. The enrichment of Proteobacteria is often associated with gut dysbiosis and inflammation (14), reinforcing the disruptive effects of *S. typhimurium* infection on the microbial community. The reduction in beneficial taxa and expansion of potentially harmful ones may contribute to the pathophysiological consequences of infection, including inflammation and barrier dysfunction.

Figure 4B through D illustrate the LEfSe cladograms for gut microbiota composition on day 2 after infection with Δ*hcp1*, Δ*hcp2*, and Δ*hcp3* strains compared to wild-type *S. typhimurium*, respectively. Compared to Fig. 4A, the differential taxa identified in these groups were fewer, suggesting that deleting any single *hcp* gene does not completely abolish the functionality of the T6SS. However, each *hcp* gene appears to have specific contributions to the microbial shifts mediated by *S. typhimurium*. In the Δ*hcp1*-infected group (Fig. 4B), significant reductions in Staphylococcaceae and Enterococcaceae were observed, which is in line with our agar plate results (Fig. 1E), while the relative abundance of Bacteroidetes, Proteobacteria, and *Butyricicoccus* significantly increased. These changes are also consistent with the patterns shown in Fig. 3A, highlighting the critical role of *hcp1* in enhancing the functional output of the T6SS. Figure 4E through G illustrates the LEfSe cladograms for day 5 after infection as well.

We also performed the Wilcox test and listed the results in Table 1 (upregulated taxa) and Table 2 (downregulated taxa). Similarly, compared to the wild-type strain, infections with Δ*hcp1*, Δ*hcp2*, and Δ*hcp3* mutant strains resulted in distinct patterns of bacterial taxa alterations. While some bacterial taxa were commonly affected across the mutants, such as *Butyricicoccus* and *Enterococcus*, others exhibited distinct trends depending on the specific *hcp* gene disrupted. The differential impact of *hcp1*, *hcp2*, and *hcp3* deletions on gut microbial composition suggests a nuanced regulatory mechanism in the *S. typhimurium* T6SS. This functional divergence may be attributed to several reasons: each *hcp* may serve as a scaffold for specific effector proteins, which target different bacterial competitors or host cells. Temporal variations in *hcp* expression may reflect their roles at different stages of infection. For example, *hcp1*’s pronounced effects on Firmicutes at day 2 may facilitate early colonization, whereas *hcp3*’s impact on Proteobacteria at day 5 could help maintain competitive dominance during later infection stages. In short, while many bacteria deploy multiple complementary T6SS systems to adapt to diverse environments, *Salmonella typhimurium* achieves similar versatility through the functional differentiation of its single T6SS system, mediated by the three *hcp* genes.

**TABLE 1:** Bacterial taxa increased in each mutant strain compared to WT^*a*^

Genus	Δ*hcp1* D2	Δ*hcp2* D2	Δ*hcp3* D2	Δ*hcp1* D5	Δ*hcp2* D5	Δ*hcp3* D5
*Butyricicoccus*	√	√	√			
*Enterococcus*					√	
*Lachnospiracea* incertae sedis	√	√				
*Streptococcus*			√			
*Anaerotruncus*		√				
Unclassified *Clostridia*		√				
*Oscillibacter*			√	√		
*Clostridium XVIII*						√
*Clostridium XlVb*				√		
*Lactobacillus*					√	√
*Mucispirillum*				√	√	
*Salmonella*					√	
*Flavonifractor*						√

^*a*^ “√” indicates taxa significantly increased compared with WT at the corresponding time point (Wilcoxon test, *P* < 0.05).

**TABLE 2:** Bacterial taxa decreased in each mutant strain compared to WT^*a*^

Genus	Δ*hcp1* D2	Δ*hcp2* D2	Δ*hcp3* D2	Δ*hcp1* D5	Δ*hcp2* D5	Δ*hcp3* D5
*Enterococcus*	√	√	√			
*Staphylococcus*	√		√	√		
*Streptococcus*	√					
Unclassified *Clostridia*						√
*Acinetobacter*			√			
*Odoribacter*			√			
*Prevotella*			√			
*Erysipelotrichaceae* incertae sedis				√		
*Jeotgalicoccus*				√	√	√
*Lactobacillus*				√		
*Acetatifactor*					√	
*Enterorhabdus*					√	

^*a*^ “√” indicates taxa significantly decreased compared with WT at the corresponding time point (Wilcoxon test, *P* < 0.05).

**Fig 5:**
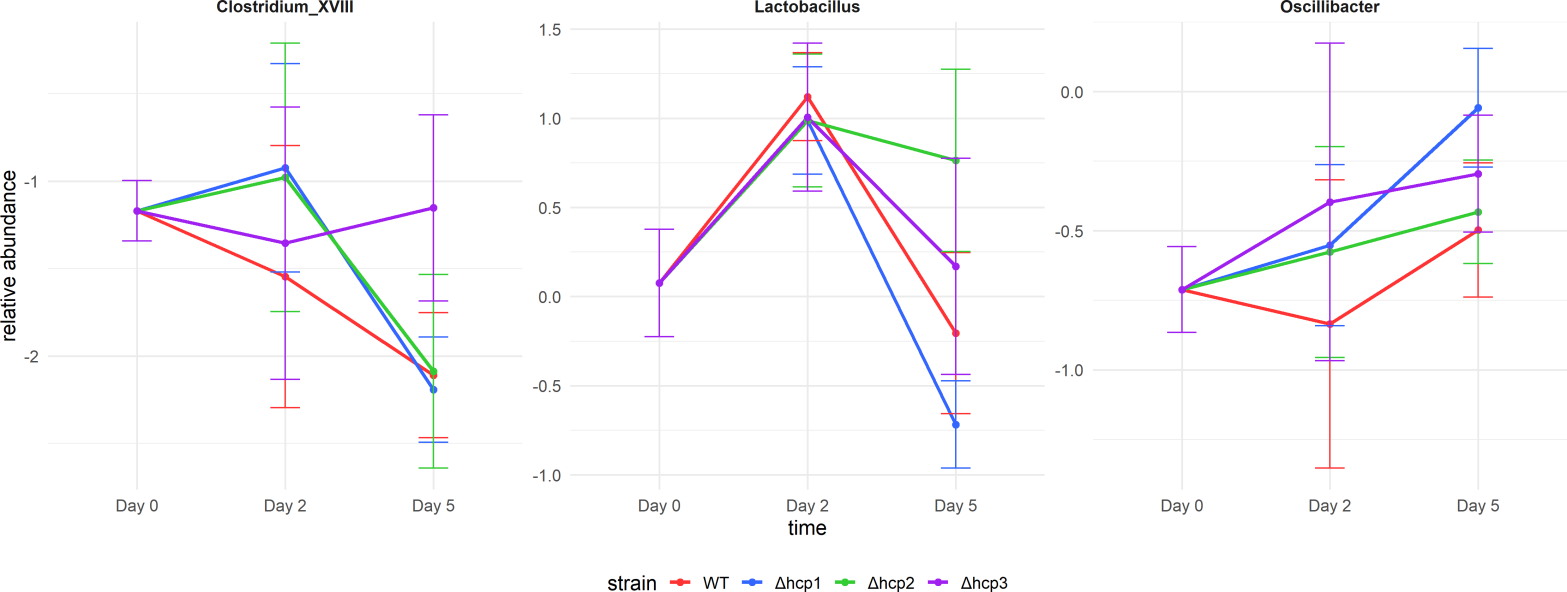
Temporal abundance trajectories of *Clostridium XVIII*, *Lactobacillus*, and *Oscillibacter*.

A two-way ANOVA analysis was also performed to assess the main effects of time, strain type (wild-type, Δ*hcp1*, Δ*hcp2*, and Δ*hcp3*), and their interaction effects on genus-level microbial composition. The statistical results are summarized in Table 3. *Lactobacillus* showed significant effects in both strain main effects and strain-time interaction effects, while *Clostridium XVIII* exhibited strong strain-time interaction effect. Fig. 5 illustrates the temporal abundance trajectories of typical genera across all groups.

**TABLE 3:** AVONA results highlighting the strain-specific roles of hcp genes in shaping the gut microbiota

Genus	Term	Statistic	*P* value	FDR	Shapiro *P*	Levene *P*
*Lactobacillus*	Strain main effect	8.904877	6.43E-05	0.001222	0.445085	0.642276
*Lactobacillus*	Time main effect	98.90433	5.58E-14	1.06E-12	0.445085	0.642276
*Lactobacillus*	Strain-time interaction effect	9.678503	3.04E-05	0.000577	0.445085	0.642276
*Clostridium XlVa*	Time main effect	18.4257	7.07E-05	0.000168	0.927565	0.482694
*Bacteroides*	Time main effect	23.60427	9.92E-06	3.77E-05	0.661796	0.27424
*Saccharibacteria* genera incertae sedis	Time main effect	23.09529	1.20E-05	3.79E-05	0.211584	0.05045
*Prevotella*	Time main effect	74.96207	6.47E-12	6.15E-11	0.774311	0.126609
*Erysipelotrichaceae* incertae sedis	Time main effect	9.750962	0.002835	0.004898	0.585627	0.388601
*Oscillibacter*	Time main effect	9.24313	0.003591	0.005685	0.299891	0.318144
*Butyricicoccus*	Time main effect	16.24362	0.00017	0.000358	0.897057	0.26728
*Clostridium XVIII*	Time main effect	20.59384	3.05E-05	8.28E-05	0.066666	0.507185
*Clostridium XVIII*	Strain-time interaction effect	4.827955	0.00465	0.044176	0.066666	0.507185
Unclassified *Lachnospiraceae*	Time main effect	26.134	4.00E-06	2.20E-05	0.062531	0.772768
Unclassified *Clostridiales*	Time main effect	14.42923	0.000361	0.000685	0.511681	0.553175
Unclassified *Bacteroidales*	Time main effect	5.358012	0.024318	0.035542	0.76558	0.570343
Unclassified *Coriobacteriaceae*	Time main effect	25.7207	4.63E-06	2.20E-05	0.716026	0.175363

### PICRUSt functional prediction

**Fig 6:**
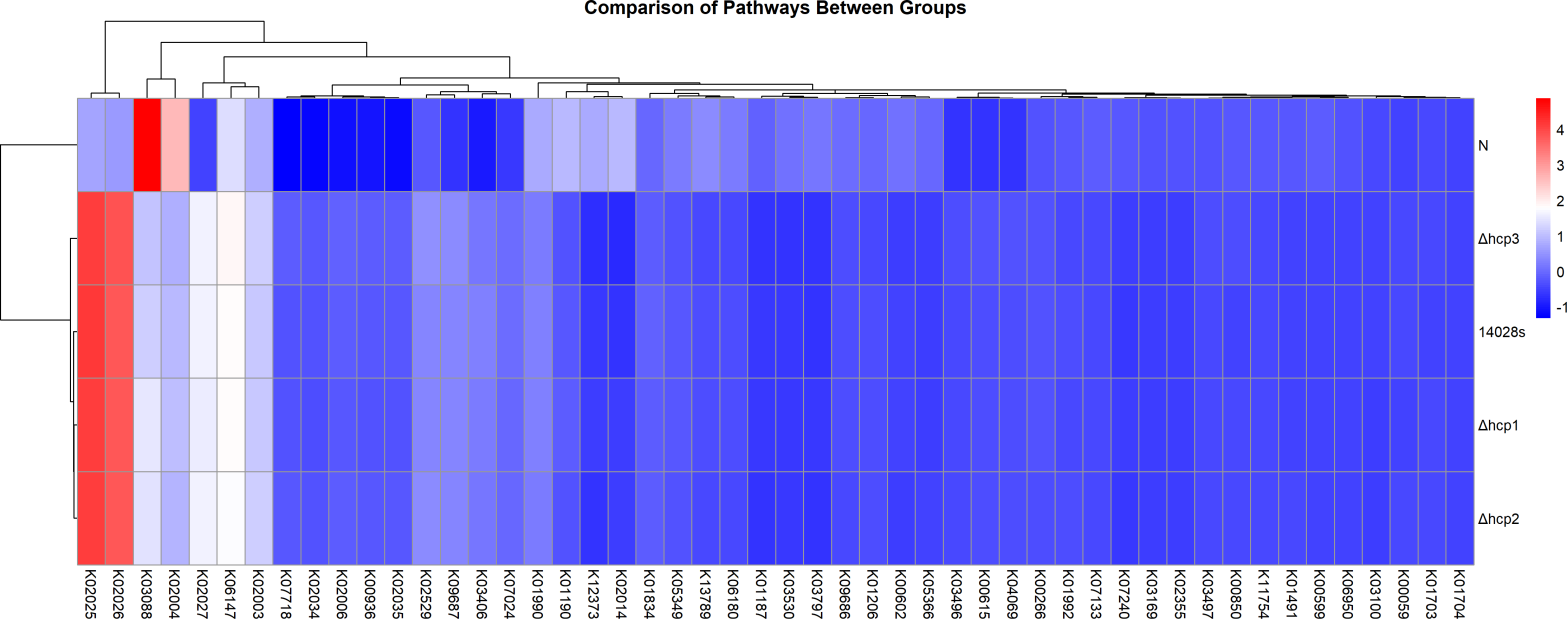
Heatmap of predicted functions before and 2 days after infection with the wild-type (WT) and its three hcp mutant strains. Infection activated the functional categories K02025 and K02026.

Figure 6 illustrates the heatmap of predicted functions in the gut microbiota before and 2 days after infection with the wild-type (WT) and its three *hcp* mutant strains. Notably, the activation of the functional categories K02025 and K02026 was inferred after infection. K02025 encodes acyl-CoA dehydrogenase, an enzyme critical for the β-oxidation of fatty acids. This pathway facilitates the breakdown of long-chain fatty acids into acetyl-CoA, which serves as a substrate for the tricarboxylic acid (TCA) cycle and is essential for energy production. Similarly, K02026 encodes short-chain acyl-CoA dehydrogenase, an enzyme specializing in the metabolism of short-chain fatty acids (SCFAs), which are key energy sources for colonic epithelial cells and play a significant role in maintaining gut health and modulating the immune response. As shown in Fig. 3, infection with *S. typhimurium*, particularly the WT strain, led to a notable increase in Firmicutes, which are prolific producers of SCFAs. The enrichment of Firmicutes likely contributes to an elevated flux of fatty acids within the gut, stimulating β-oxidation pathways to metabolize these substrates. It is, thus, reasonable that the infection induces a shift in host metabolism to prioritize energy production and repair mechanisms. Acyl-CoA dehydrogenases appear to play an essential role in this metabolic responding, potentially enabling the host to utilize microbial SCFAs and long-chain fatty acids for energy under the stress of infection.

While this study reveals the distinct ecological impacts of individual *hcp* genes, the exact mechanism and consequent effects on host immunity remain an open question. Future studies including identifying specific effector proteins and correlating these specific microbiota profiles with host inflammatory responses (e.g., via cytokine profiling, histopathological examinations) will be crucial to fully understand the functional significance of T6SS-microbiota interactions.

### Limitations

Although we collected both cecal and colonic contents from each mouse, effectively doubling the number of biological replicates for microbial community analysis, the baseline sample size remains moderate.

### Conclusion

In summary, we explored the impact of *S. typhimurium* and its type VI secretion system (T6SS) on the gut microbiota of BALB/c mice, focusing on the roles of three *hcp* genes (*hcp1*, *hcp2*, and *hcp3*). *S. typhimurium* infection disrupts gut microbiota richness, as evidenced by reduced alpha diversity, while the evenness of the microbiota remains unaffected. Notably, infection with *S. typhimurium* caused significant gut dysbiosis within 2 days, which gradually began to recover by day 5, reflecting the dynamic nature of microbiota responding during infection. The data also revealed that the deletion of each *hcp* genes led to distinct changes in the microbial community, demonstrating that these Hcp proteins contribute non-redundantly to T6SS function. These distinct functions could influence interactions with specific microbial taxa, thereby potentially shaping the dynamics of gut colonization and affecting the microbial balance during infection. The predicted functions of the gut microbiota were also addressed. These insights could inform future research into microbiota-mediated pathogenesis and contribute to the development of attenuated live vaccines or therapeutic strategies that exploit microbiome modulation.

## Data Availability

The data are available at Figshare: https://doi.org/10.6084/m9.figshare.29434379.

## SUPPLEMENTAL MATERIAL

Supplemental figures spectrum.02225-25-s0001.docx Fig. S1 to S8.

Supplemental tables spectrum.02225-25-s0002.xlsx Tables S1 and S2.

ASM does not own the copyrights to Supplemental Material that may be linked to, or accessed through, an article. The authors have granted ASM a non-exclusive, world-wide license to publish the Supplemental Material files. Please contact the corresponding author directly for reuse.
