# Supplementary material for: Divergent roles of hcp genes in Salmonella typhimurium T6SS shape gut microbiota dysbiosis during infection
Source: Microbiol Spectr. 2026 Feb 27;14(4):e02225-25. doi: 10.1128/spectrum.02225-25 (PMC13055400; doi:10.1128/spectrum.02225-25)
Supplement: Supplemental figures — Fig. S1 to S8. [file spectrum.02225-25-s0001.docx]

**Divergent Roles of *hcp* Genes in *Salmonella* *typhimurium* T6SS Shape Gut Microbiota Dysbiosis during Infection**

Jia Liu^1^†, Jiao Liu^2^†, Chengjie Feng^1^, Qinghua Zou^1^*

^1^ Department of Microbiology and Infectious Disease Center, School of Basic Medical Sciences, Peking University Health Science Center, Beijing, 100191, China

^2^ Center of Medical and Health Analysis, Peking University Health Science Center, Beijing, China

*** Corresponding author contact information**

Qinghua Zou, Ph.D.

Department of Microbiology and Infectious Disease Center, School of Basic Medical Sciences, Peking University Health Science Center

Address: 38 Xueyuan Road, Haidian District, Beijing 100191, China

Phone: 86-10-82805070

E-mail: [zouqinghua@bjmu.edu.cn](mailto:zouqinghua@bjmu.edu.cn)

† Jia Liu and Jiao Liu contributed equally to this work.


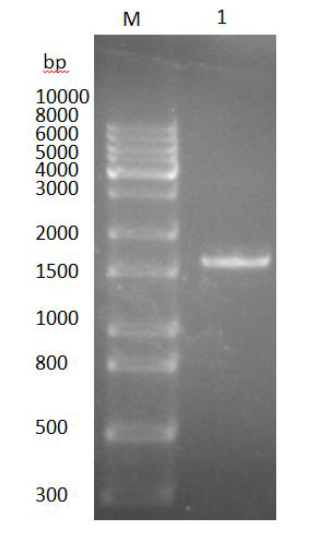


Figure S1. Agarose gel electrophoresis of PCR products. M: marker; 1: homologous fragment PCR product.


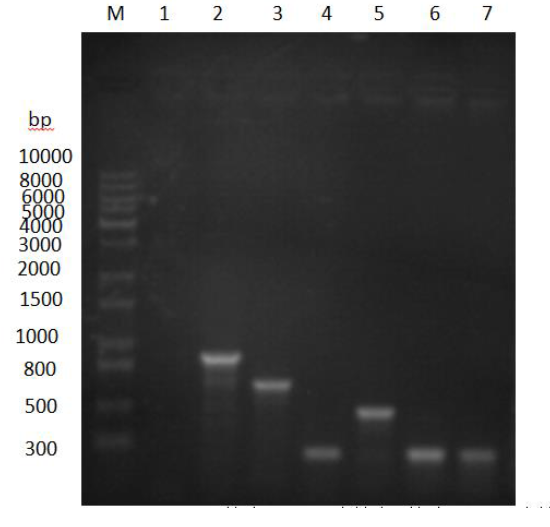


Figure S2. Verification of resistance gene elimination in *hcp* knockout strains.

Note: M, marker; 1, negative control; 2, positive control for *hcp1* (undeleted); 3, positive control for *hcp3* (undeleted); 4, 6, and 7 correspond to PCR results of Δ*hcp3*, Δ*hcp1*Δ*hcp3*, and Δ*hcp2*Δ*hcp3* after deletion of the antibiotic-resistance cassette; 5 corresponds to the PCR result of Δ*hcp1*Δ*hcp2* after antibiotic-resistance cassette deletion.


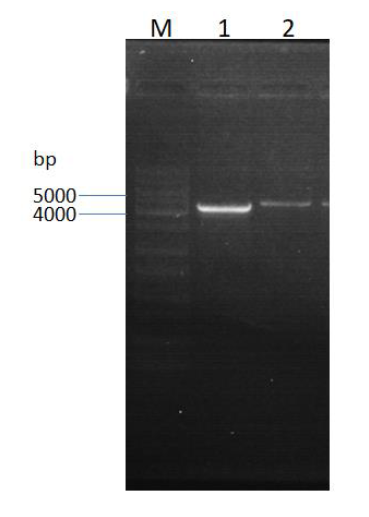


Figure S3. Agarose gel electrophoresis analysis of complementation plasmid construction.

M, marker; 1, original plasmid pTrc99A; 2, complementation plasmid pTrc99A-*hcp3*.


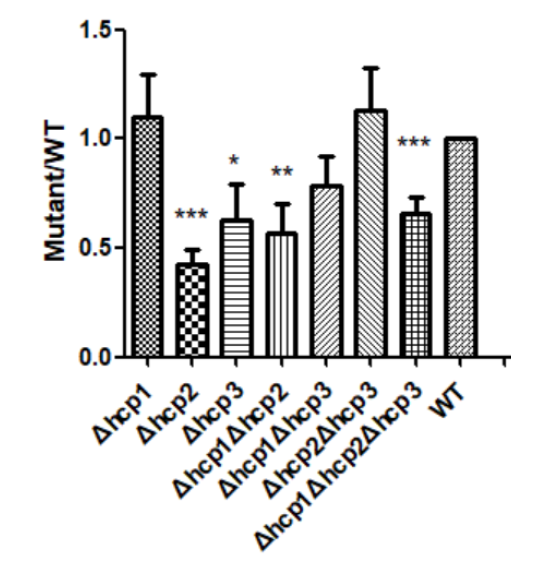


Figure S4. Amoeba adhesion assay.


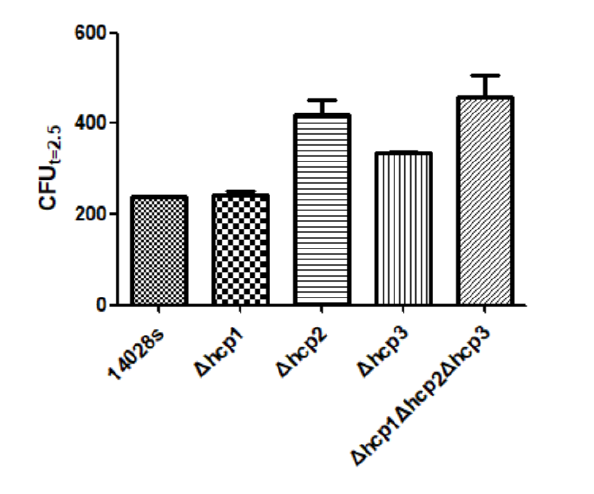


Figure S5. Bacterial resistance to amoeba phagocytosis assay.

Figure S6. Body weight of mice infected with wild-type *S. typhimurium* and its three *hcp* mutant strains.


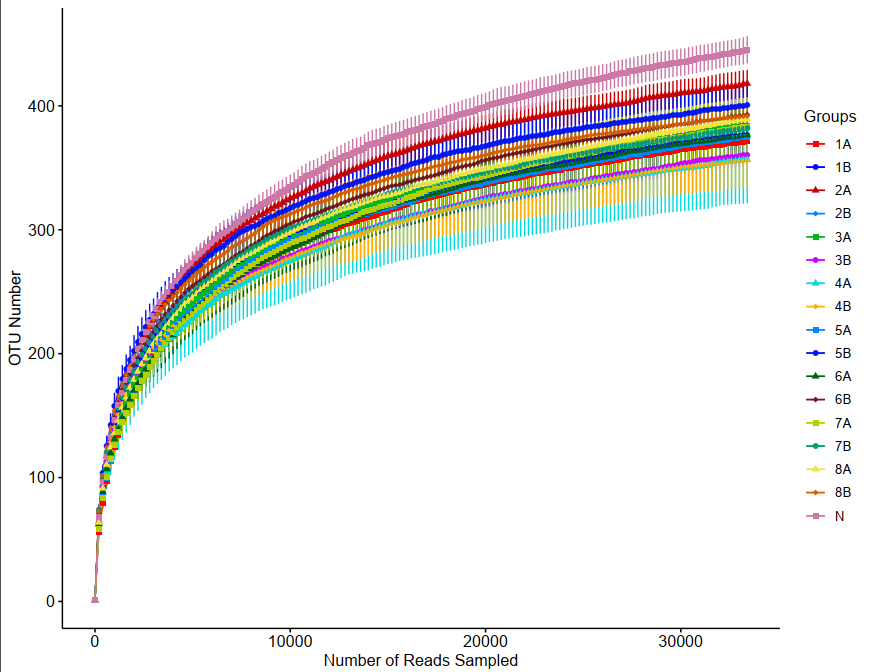


Figure S7. Rarefaction Curve of the 16S rRNA sequencing data.


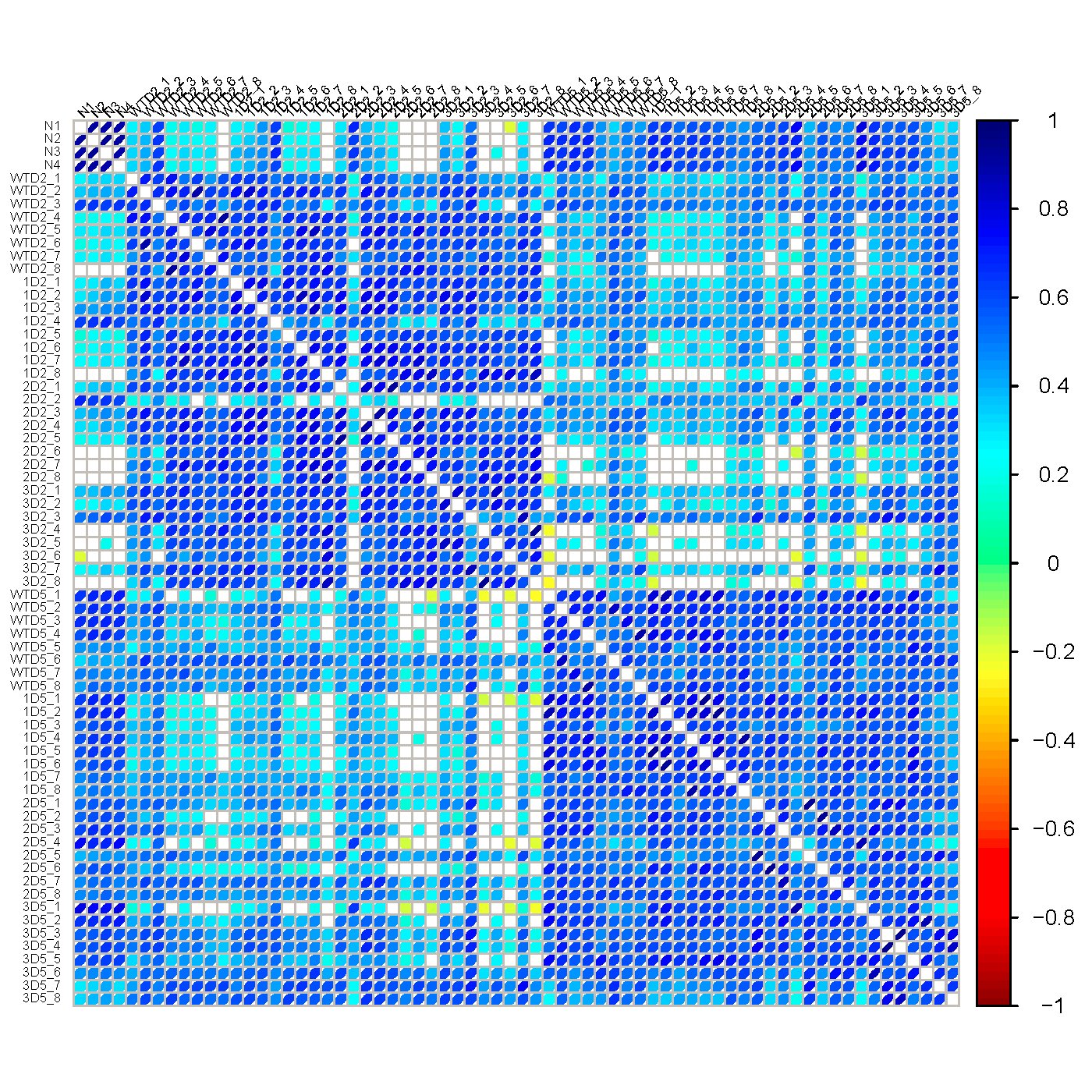


Figure S8. Samplecorrplot indicating the similarity and relativity among samples.
